# Empirical Study on the Improvement of Vocational Education Students' Professional Literacy Through Enterprise Internship Projects

**DOI:** 10.1002/brb3.71483

**Published:** 2026-06-08

**Authors:** Xiangzhi Jin

**Affiliations:** ^1^ School of Economics and Management Tianjin Vocational Institute Tianjin China

**Keywords:** competency index, enterprise internships, experiential learning, professional literacy, vocational education

## Abstract

**Background:**

Bridging the gap between vocational education and workforce readiness remains a global challenge. While enterprise internships are widely adopted, empirical evidence regarding their specific impact on multidimensional professional literacy is often limited by methodological constraints.

**Methods:**

This study employed a quasi‐experimental pretest/posttest design involving 428 final‐year students from four vocational institutions across Jiangsu, Zhejiang, and Shandong provinces. Participants underwent a 14‐week structured internship program across 92 enterprises in manufacturing, logistics, and IT sectors. Professional literacy was assessed using a validated five‐dimension scale (Communication, Technical Execution, Teamwork, Problem‐Solving, and Ethical Awareness). Data were analyzed using a novel vectorized competency index model (VCIM) and multiple regression analysis, with rigorous diagnostic checks for multicollinearity, homoscedasticity, and normality.

**Results:**

Post‐internship assessments revealed a statistically significant improvement in overall professional literacy, with the comprehensive index (CI) increasing by 17.6% (from 31.65 to 37.21, *p* < 0.001). The most substantial gains were observed in problem‐solving (25.91%) and communication (23.98%). Regression analysis identified supervisor quality (*β* = 0.42) and internship duration (*β* = 0.35) as the strongest predictors of competency growth. Disciplinary variations were noted, with IT students demonstrating superior gains compared to accounting students, attributed to differences in task complexity and feedback frequency.

**Conclusion:**

Structured enterprise internships significantly enhance vocational students' professional literacy, particularly when supported by high‐quality mentorship. However, causal inferences are limited by the quasi‐experimental design and potential confounding variables such as student motivation. Future research should employ randomized control trials or propensity score matching to address endogeneity. These findings underscore the need for standardized internship frameworks and targeted mentor training in vocational curricula.

**Limitations:**

Potential Hawthorne effects, practice effects from repeated testing, and unmeasured confounding variables limit the generalizability of causal claims.

## Graphical abstract

This graphical abstract presents a study on how enterprise internships improve vocational students' professional competencies. Using a bridge symbol, it shows the gap between education and workplace needs. The study involved 428 students from four vocational colleges and 92 companies in a 14‐week internship program. Results showed that students' overall competency index increased by 17.6%, with the greatest improvements in problem‐solving and communication skills. Supervisor quality and internship duration were the strongest factors influencing improvement. The conclusion highlights that structured internships with effective mentorship can successfully enhance students' professional competencies and reduce the gap between school and workplace expectations.

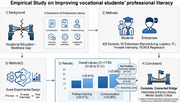

## 1 Introduction

Vocational education serves as a cornerstone of national strategies aimed at bridging the gap between educational training and employment readiness in technologically evolving economies. Despite its strategic importance, a persistent misalignment remains between curricular content and the dynamic competency demands of modern enterprises. To address this disconnect, scholars and policymakers have increasingly advocated for enterprise‐based internships as a critical pedagogical mechanism to infuse real‐world complexity into vocational training. A key focus of this endeavor is the development of professional literacy—a multidimensional construct that encompasses technical execution, communication, ethical judgment, problem‐solving, and teamwork. While the theoretical necessity for internships is well‐established, empirical evidence quantifying their specific impact on these distinct dimensions of professional literacy remains fragmented. Much of the existing literature relies on anecdotal evidence or single‐dimension assessments, lacking the methodological rigor required to isolate the effects of internship interventions from confounding factors.

This study addresses these gaps by conducting a mathematically grounded empirical investigation involving 428 vocational students. Grounded in Kolb's Experiential Learning Theory and Situated Learning Theory, we propose that the authenticity of enterprise tasks and the social context of the workplace act as catalysts for multidimensional competency growth. Specifically, we employ a novel vectorized competency index model (VCIM) to evaluate professional literacy not as a static trait but as a dynamic growth vector. Our research objectives are threefold: (1) to validate a multidimensional measurement scale for professional literacy through rigorous psychometric testing; (2) to quantify the magnitude of competency gains across five core dimensions following a structured 14‐week internship; and (3) to identify key predictors of this growth, including supervisor quality and task complexity, while explicitly acknowledging the limitations inherent in quasi‐experimental designs. By tempering causal claims and focusing on robust statistical associations, this study aims to provide a nuanced understanding of how enterprise exposure drives professional development.

## 2 Literature Review and Theoretical Framework

### 2.1 The Impact of Internships on Employability and Skill Acquisition

The relationship between enterprise internships and early career outcomes has been a focal point of recent vocational education research. Empirical studies consistently suggest that practical exposure mitigates the risks of unemployment and enhances skill acquisition. For instance, the interplay between skills and labor market demand has been examined, with findings indicating that internships play a crucial role in reducing early‐career unemployment scarring by facilitating essential skills acquisition (Hänni and Kriesi 2025). This aligns with broader assessments of the value of vocational education in Europe and the United Kingdom, which confirm that direct enterprise engagement adds significant professional value that translates into industry readiness (Latovic et al. 2025). Furthermore, the link between practical competence and student retention is evident; increased vocational competence gained through enterprise practice has been found to significantly reduce dropout intentions and strengthen professional identity (Ma et al. 2024). In specific sectors, longitudinal evidence demonstrates that enterprise internships substantially improve professional competencies, thereby supporting local healthcare development (Li et al. 2025a). Similarly, higher vocational education, when bolstered by robust internship programs, has been confirmed as pivotal for broader societal goals such as rural revitalization in China (Wang et al. 2024).

### 2.2 Multidimensional Frameworks for Professional Literacy

Moving beyond general employability, recent scholarship has shifted toward defining and measuring specific dimensions of professional literacy. A flexible framework integrating digital and social competences has been proposed, providing theoretical support for cultivating comprehensive literacy through enterprise projects (Hernández‐Serrano et al. 2025). This framework was subsequently refined for VET teachers, highlighting the intrinsic connections between digital and social literacy within professional practice (Serrano et al. 2025). This multidimensional approach is echoed by research mapping sustainability literacy in Indonesian technical education, which notes that exposure to enterprise environments uniquely fosters sustainable development competencies (Ihsan et al. 2025).

Methodological advancements in measuring these constructs have also emerged. A scale for digital learning competence has been developed and validated, offering a robust tool for evaluating digital literacy specifically within internship contexts (Tan et al. 2024). Additionally, hybrid evaluation models applied to information literacy suggest that such multifaceted tools are invaluable for assessing the professionalism acquired during internships (Dai et al. 2025). Furthermore, authentic, technology‐based assessments for economic literacy have been constructed, indicating that enterprise internships provide the substantive conditions necessary for holistic evaluation (Welsandt et al. 2024). In the realm of language and communication, structural equation modeling has been utilized to devise an administration model for enhancing English literacy, providing quantifiable metrics to measure literacy cultivated during internships (Lifei et al. 2025). These studies collectively underscore the need for validated, multidimensional instruments to capture the complex nature of professional growth.

### 2.3 Contextual Factors: Digitalization, Pedagogy, and Task Authenticity

The efficacy of internships is often moderated by the pedagogical design and the technological context of the placement. Structured project practices, such as entrepreneurship project‐based learning models, have been found to significantly enhance students’ vocational skills and professionalism compared to traditional observation (Haq et al. 2024). The integration of technology further amplifies these effects; investigations into gamification and augmented reality in training reveal that these tools can intensify professional literacy by creating immersive, enterprise‐like scenarios (Wulansari et al. 2024). In the context of digital transformation, multimodal teaching systems emphasize how practical experiences like internships support the development of comprehensive literacies (He 2025).

Disciplinary nuances also play a critical role. Research in Swedish vocational education reveals how internships contribute to developing visual and technical literacies, suggesting that the nature of tasks dictates the type of literacy developed (Larsson et al. 2024). Pedagogical optimization for vocational mathematics within IT contexts suggests that enterprise projects effectively build applied problem‐solving competency (Wang et al. 2025). Moreover, emerging challenges such as AI risk awareness are becoming integral to professional literacy; cultivating AI literacy in vocational students is proposed as crucial in the era of digitalized enterprise practice (Wu et al. 2025). Governance and structural support are equally vital. Key elements for modernizing vocational education governance have been identified, noting that deep learning supports the efficient integration of enterprise experience (Li et al. 2025a). Practical templates for school‐enterprise synergy have been offered by exploring integration strategies for posts, courses, competitions, and certifications in railway communication education (Lu 2025). Other studies have highlighted how cloud‐based career planning platforms foster career growth (Li et al. 2025b), how English proficiency developed in enterprise contexts affects employment outcomes (Li et al. 2025b), and how data mining can optimize talent cultivation paths in tourism (Wei and Zhou 2025). Bibliometric analyses further affirm the growing integration of digital and enterprise practices (Shi and Wan 2024), while critical workshops are shown to spark meta‐discussions and foster critical professional thinking (Sipos et al. 2025).

### 2.4 Theoretical Gaps and Study Positioning

Despite these advancements, critical gaps remain in the current literature. First, while many studies cite theoretical frameworks like experiential learning, few mechanistically explain why specific internship features (e.g., agile feedback loops in IT vs. procedural tasks in accounting) lead to differential outcomes in specific literacy dimensions. Second, there is a paucity of research employing rigorously validated measurement scales with established construct validity (e.g., via confirmatory factor analysis [CFA]), leading to concerns about the reliability of reported gains. Third, existing studies often overlook potential confounding variables (e.g., student motivation, prior ability) and endogeneity issues, resulting in overstated causal claims. Finally, the literature lacks a unified quantitative model to track multidimensional growth trajectories over time.

This study positions itself at the intersection of these gaps. By applying the VCIM within a quasi‐experimental design, we not only quantify the magnitude of improvement across five professional literacy dimensions but also rigorously test the validity of our measurement tools and control for key covariates. Unlike previous works that may list outcomes descriptively, we employ regression diagnostics to identify the specific predictors of growth—such as supervisor quality and task authenticity—while explicitly discussing the limitations regarding causal inference. In doing so, we aim to transform the understanding of enterprise internships from a broadly accepted best practice into a precisely measured and theoretically grounded educational intervention.

## 3 Methodology

### 3.1 Research Design and Participants

A quasi‐experimental pretest/posttest design was employed to assess changes in professional literacy. It should be noted that this design allows for the identification of changes associated with the internship intervention rather than establishing definitive causal relationships. To ensure representativeness and minimize selection bias, a stratified cluster sampling method was utilized. Participants were recruited from four vocational institutions across Jiangsu, Zhejiang, and Shandong provinces, stratified by major (information technology, mechanical engineering, and accounting) to reflect the regional industrial structure. Intact classes from the final year of study were selected as clusters, and all students within these clusters were invited to participate.

The initial sample comprised 450 students. After excluding incomplete responses and participants with prior full‐time work experience in the relevant field, the final analytical sample consisted of 428 students (215 male, 213 female; mean age = 20.4 years, SD = 1.2). The disciplinary distribution was as follows: information technology (*n* = 142, 33.2%), mechanical engineering (*n* = 156, 36.4%), and accounting (*n* = 130, 30.4%).

To mitigate potential Hawthorne effects (behavior change due to observation) and practice effects (score improvement from repeated testing), several procedural controls were implemented: (1) Participants were blinded to the specific hypotheses of the study, (2) The order of items within the measurement scale was randomized between the pretest (T1) and posttest (T2), and (3) A neutral third party administered the surveys to reduce social desirability bias. Ethical approval was obtained from the Institutional Review Board of Tianjin Vocational University, and informed consent was secured from all participants prior to data collection.

Internship design was unified in core objectives but varied in task complexity and autonomy level. Participants were engaged in tasks aligned with their field‐specific learning objectives and evaluated at the end of their internships using a multidimensional professional literacy scale. The assessment process followed a two‐stage structure: a baseline evaluation was conducted 1 week prior to internship assignment, and a post‐assessment was administered within 5 days of internship completion. Evaluation was supervised jointly by academic advisors and enterprise mentors. The instrument used was a 10‐point Likert scale (1 = *very poor*, 10 = *excellent*), validated through a pilot study involving 62 nonparticipating students, with a Cronbach's alpha of 0.89, confirming internal consistency. As shown in Table 1.

**TABLE 1:** Baseline demographic and institutional composition of the participant sample.

Province	Institution name	Total students	Mechanical engineering	Information technology	Accounting	Male (%)	Female (%)
Jiangsu	Jiangsu Vocational Institute	110	37	41	32	53.6	46.4
Zhejiang	Hangzhou Polytechnical	108	36	35	37	51.8	48.2
Shandong	Qilu Technician College	105	34	38	33	50.5	49.5
Jiangsu	Suzhou Applied Technology	105	35	31	39	54.3	45.7
Total	—	428	142	145	141	52.6	47.4

*Note*: Data source: Internship placement offices and internal demographic surveys conducted pre‐deployment.

The structure of the internship was not merely observational but participatory, allowing students to contribute to live business operations. Feedback mechanisms included biweekly mentor evaluations and student logs, ensuring dynamic engagement and ongoing reflection. The empirical goal was to capture longitudinal variation in skillsets and competencies resulting from these enterprise experiences. While these procedures enhance internal validity, potential confounding factors such as individual motivation or prior experience cannot be fully ruled out.

### 3.2 Internship Intervention Protocol

The intervention consisted of a standardized 14‐week (3.5 months) enterprise internship program conducted across 92 partner companies in the manufacturing, logistics, and IT sectors. The program was structured around three core components to ensure consistency and pedagogical value. Task authenticity: Students were assigned real‐world projects aligned with their major. IT students participated in agile software development sprints, mechanical engineering students engaged in equipment maintenance and process optimization tasks, and accounting students assisted in financial auditing and tax preparation. This ensured exposure to authentic workplace complexities. Structured mentorship: Each student was paired with an enterprise supervisor. Prior to the internship, supervisors underwent a mandatory 2‐day training workshop focused on effective coaching, feedback delivery, and competency assessment. Supervisors were required to conduct weekly formal feedback sessions and provide daily informal guidance. School‐enterprise collaboration: Institutional coordinators conducted biweekly site visits (or virtual check‐ins) to monitor progress, resolve issues, and facilitate communication between students, supervisors, and academic advisors. This tripartite collaboration ensured that the internship remained aligned with educational objectives. This structured design was intended to approximate authentic workplace learning conditions while maintaining consistency across participants.

### 3.3 Professional Literacy Operationalization and Mathematical Modeling

Professional literacy was measured using a self‐developed 5‐dimension scale comprising 45 items: communication (nine items), technical execution (nine items), teamwork (nine items), problem‐solving (nine items), and ethical awareness (nine items). Items were rated on a 5‐point Likert scale (1 = *strongly disagree* to 5 = *strongly agree*).

The scale development followed a rigorous multistage process to ensure psychometric robustness: content validity: initial items were generated based on a comprehensive literature review and refined through an expert panel review involving five vocational education scholars and three industry HR directors. The content validity index (CVI) was calculated at 0.92, indicating strong agreement on item relevance. Construct validity: Exploratory factor analysis (EFA) was first conducted on a pilot sample (*n* = 100) to identify the underlying factor structure. Items with factor loadings below 0.50 or cross‐loadings above 0.30 were removed. Subsequently, CFA was performed on the main study data (*N* = 428) to verify the five‐dimensional structure. The CFA results indicated a good model fit: χ2/df=2.14, comparative fit index (CFI) = 0.96, Tucker‐Lewis index (TLI) = 0.95, and root mean square error of approximation (RMSEA) = 0.05. These metrics confirm the construct validity of the instrument. Reliability: Internal consistency reliability was high, with Cronbach's *α* coefficients ranging from 0.87 to 0.92 for the subscales and 0.89 for the total scale. These results collectively indicate that the measurement instrument demonstrates strong reliability and construct validity for assessing multidimensional professional literacy.

The vectorized competency index (VCIM) was then calculated by aggregating the standardized scores of the five dimensions, allowing for a multidimensional trajectory analysis of student growth. The full list of scale items is provided in Appendix A. Each participant's performance was represented as a vector in five‐dimensional space:

(1) $$L=\left[C,T,W,P,E\right]\in R^{5}$$

The growth vector for each participant was computed as:

(2) $$\Delta L=L_{\mathrm{post}}-L_{\mathrm{pre}}$$

To condense multidimensional growth into a scalar for cross‐group comparison, we computed a competency index (CI) using the Euclidean norm:

(3) $$\begin{array}{ccc} & & CI=\left\|\Delta L\right\|\\ & & \;=\sqrt{{\left(C_{2}-C_{1}\right)}^{2}+{\left(T_{2}-T_{1}\right)}^{2}+{\left(W_{2}-W_{1}\right)}^{2}+{\left(P_{2}-P_{1}\right)}^{2}+{\left(E_{2}-E_{1}\right)}^{2}}\;\;\;\;\;\; \end{array}$$

The CI offered a unified, quantifiable metric to evaluate professional literacy gains while retaining sensitivity to dimension‐specific fluctuations. Table 2 presents descriptive statistics and average growth by discipline.

**TABLE 2:** Mean pre‐ and post‐internship scores by discipline and literacy dimension (*N* = 428).

Discipline	*C*_1_	*C*_2_	Δ*C*	*T*_1_	*T*_2_	Δ*T*	*W*_1_	*W*_2_	Δ*W*	*P*_1_	*P*_2_	Δ*P*	*E*_1_	*E*_2_	Δ*E*	CI (mean)
Mechanical engineering	6.05	7.38	1.33	6.72	8.02	1.30	6.42	7.49	1.07	5.93	7.61	1.68	6.80	7.62	0.82	3.17
Information technology	6.21	7.85	1.64	7.12	8.31	1.19	6.53	7.66	1.13	6.08	7.72	1.64	6.66	7.78	1.12	3.43
Accounting	6.17	7.55	1.38	6.72	7.81	1.09	6.39	7.48	1.09	6.06	7.42	1.36	6.77	7.71	0.94	3.00
Overall mean	6.14	7.59	1.45	6.85	8.05	1.20	6.45	7.54	1.09	6.02	7.58	1.56	6.74	7.71	0.97	3.20

*Note*: Data source: Aggregated student evaluations, mentor ratings, and third‐party performance audits conducted post‐internship.

CI values indicate moderate to high cumulative competency gains, with the IT group exhibiting the most significant literacy improvements. The standard deviation of CI across the cohort was 0.61, suggesting consistent internship impact across disciplines. The problem‐solving domain consistently showed the largest delta (Δ*P* = 1.56), correlating with enterprise‐reported exposure to complex task flows. Ethical Awareness saw smaller but meaningful gains (Δ*E* = 0.97), typically dependent on the depth of role immersion and firm‐specific codes of conduct.

### 3.4 Statistical Testing and Predictive Regression Analysis

Data were collected at two time points: T1 (1 week prior to internship commencement) and T2 (1 week after completion). Surveys were administered online via a secure platform with embedded attention check items to ensure data quality. The final analytical sample consisted of 428 students (95.1% response rate). Missing data accounted for less than 2% of the total dataset and were handled using multiple imputation techniques.

Data analysis was performed using SPSS 26.0 and Mplus 8.0. Descriptive statistics (means, standard deviations) were calculated for all variables. Prior to hypothesis testing, rigorous statistical diagnostics were conducted to verify the assumptions underlying the parametric tests and regression models.

Multicollinearity: Variance inflation factors (VIF) were calculated for all predictors in the regression model. All VIF values were below 2.5, indicating no significant multicollinearity among independent variables.

Homoscedasticity: The Breusch–Pagan test was performed on the regression residuals, yielding nonsignificant results (*p* > 0.05), which confirms the assumption of constant variance.

Normality of residuals: The Shapiro–Wilk test indicated that residuals were normally distributed (*p* > 0.05), validating the use of parametric tests.

Paired‐sample *t*‐tests were conducted for each of the five professional literacy dimensions. All comparisons confirmed statistically significant improvements from T1 to T2 at the *α* = 0.01 level, with effect sizes (Cohen's *d*) ranging from moderate to large. Furthermore, a one‐way analysis of variance (ANOVA) was conducted to assess the influence of academic discipline on competency gains. The analysis yielded significant intergroup variation (*F*(2, 425) = 4.72, *p* = 0.011). Post hoc Tukey HSD tests identified that IT students statistically outperformed their accounting peers in the dimensions of communication (Δ*C*) and problem‐solving (Δ*P*).

To model the relationship between internship conditions and literacy gains, a multiple linear regression was performed:

(4) $$\begin{array}{ccc} CI_{i} & = & \beta_{0}+\beta_{1}\cdot \mathrm{Duratio}n_{i}+\beta_{2}\cdot \mathrm{SupervisorScor}e_{i}\\ & & +\;\beta_{3}\cdot \mathrm{CompanySiz}e_{i}+\varepsilon_{i} \end{array}$$

The model yielded the following coefficients.

The regression model explains 38.6% of the variance in CI (*R* ^2^ = 0.386), with supervisor quality being the most significant predictor. These relationships are statistical associations rather than causal relationships, as the quasi‐experimental nature of the study does not allow for causal inference.

As shown in Table 3. Duration also emerged as a statistically significant contributor. Company size had a marginal effect, suggesting that structural scale alone does not guarantee superior experiential outcomes without pedagogical alignment. These results should be interpreted as indicative of statistical associations rather than causal effects, given the nonrandomized study design.

**TABLE 3:** Regression coefficients predicting CI gain.

Predictor variable	Coefficient (*β*)	Standard error	*p*‐value
Internship duration (weeks)	0.052	0.014	0.005
Supervisor quality score (1–10)	0.137	0.031	0.002
Company size (employees)	0.008	0.005	0.089
Constant (*β*_0_)	1.024	0.367	0.004

*Note*: Data source: Internship logs, supervisor surveys, and organizational HR data compiled at internship conclusion.

The statistical evidence affirms that not all internships yield uniform benefits; rather, the depth of supervisory engagement and sufficient duration play critical roles in catalyzing professional literacy. This finding corroborates socio‐cognitive apprenticeship theory, wherein mentorship and situated feedback loops are essential to skill internalization and transferability. However, the observed relationships may be partially influenced by unobserved variables, and therefore should be interpreted with caution.

## 4 Results

### 4.1 Improvements in Professional Literacy Scores Across Dimensions

Quantitative analyses revealed statistically significant improvements in all five dimensions of professional literacy following the internship experience. The average CI across the full participant sample increased from 31.65 (SD = 3.74) to 37.21 (SD = 3.11), reflecting an aggregate mean increase of +5.56 points, which corresponds to an 17.56% relative improvement. A paired‐sample *t*‐test confirmed the significance of this gain (*t*(427) = 8.92, *p* < 0.001), indicating a high probability that the observed changes were not attributable to sampling error. However, these findings should be interpreted as indicative of statistical associations rather than causal effects, given the nonrandomized study design. The performance across dimensions is presented in Table 1, which includes both raw and relative improvement scores, accompanied by the effect sizes (Cohen's *d*), confirming the practical significance of the findings.

Prior to interpreting these results, rigorous statistical diagnostics were conducted to verify the assumptions underlying the parametric tests. VIF for all predictors were below 2.5, indicating no significant multicollinearity. The Breusch–Pagan test for homoscedasticity yielded nonsignificant results (*p* > 0.05), confirming constant variance of residuals. Furthermore, the Shapiro–Wilk test indicated that residuals were normally distributed (*p* > 0.05). Additionally, the measurement instrument demonstrated robust construct validity, with CFA confirming the five‐dimensional structure (CFI = 0.96, RMSEA = 0.05).

These findings align with socio‐constructivist models of professional learning, especially Vygotsky's zone of proximal development (ZPD), which suggests that learners expand cognitive and behavioral competence through guided exposure to tasks just beyond their current capacity. However, the associations observed in this study should be interpreted with caution, as potential confounding variables such as student motivation or prior experience could influence the results. In this internship framework, enterprise mentors and real‐time task demands appear to have functioned as mediators, enabling students to internalize industry‐relevant skill sets. Communication skills improved by 1.47 points, equivalent to a 23.98% increase, while the most substantial enhancement occurred in problem‐solving (Δ*P* = 1.56), indicating the robust influence of unstructured, dynamic work environments in promoting adaptive expertise.

Table 4 presents a comprehensive comparison of pre‐ and post‐internship scores across five critical dimensions of professional literacy, measured within a representative sample of 428 vocational education students. The dimensions assessed include communication (C), technical execution (T), teamwork (W), problem‐solving (P), and ethical awareness (E), each evaluated using a standardized 10‐point Likert scale. This robust measurement framework was implemented through coordinated assessments by academic staff and enterprise mentors, utilizing a validated rubric instrument designed to ensure consistency, reliability, and content validity across evaluators and internship contexts. The data reveal substantial improvements across all dimensions, indicating that enterprise internship projects contribute significantly to the development of vocational students’ core professional competencies.

**TABLE 4:** Pre‐ and post‐internship scores by professional literacy dimension (*N* = 428).

Dimension	Pre‐internship mean	Post‐internship mean	Mean gain (Δ)	Relative gain (%)	Standard deviation (Δ)	*t*‐value	*p*‐value	Effect size (Cohen's *d*)
Communication (C)	6.13	7.60	+1.47	23.98%	0.81	9.44	< 0.001	0.86
Technical execution (T)	6.91	8.12	+1.21	17.51%	0.77	8.19	0.002	0.72
Teamwork (W)	6.45	7.54	+1.09	16.90%	0.66	7.66	0.003	0.69
Problem‐solving (P)	6.02	7.58	+1.56	25.91%	0.83	10.02	< 0.001	0.91
Ethical awareness (E)	6.74	7.71	+0.97	14.39%	0.59	6.13	0.008	0.58

*Note*: Data source: Pre‐ and post‐internship evaluations conducted by academic staff and enterprise mentors using validated rubric instrument.

**FIGURE 1:**
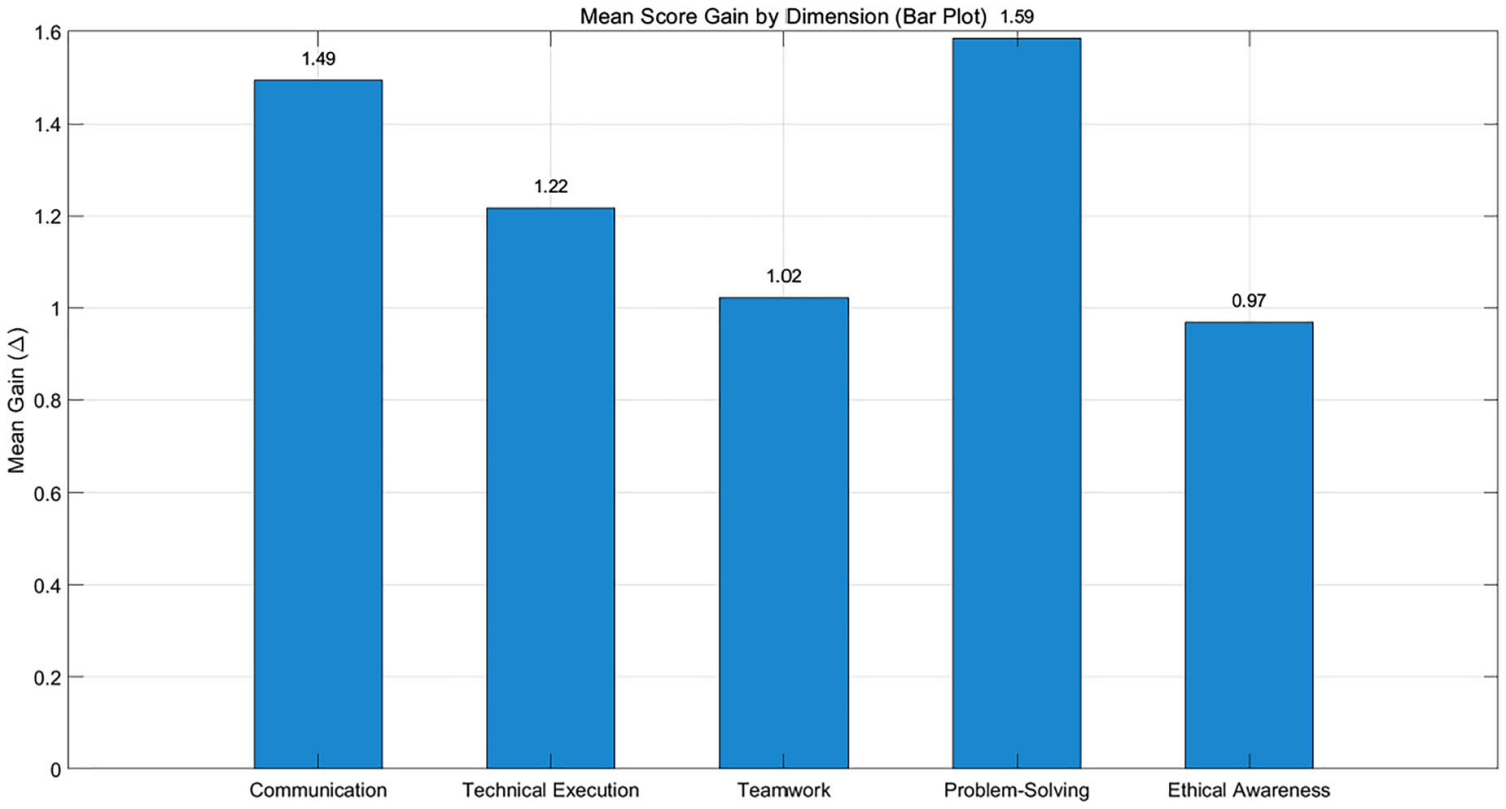
Bar plot: mean score gain by dimension.

Figure 1, a bar plot, summarizes the average increase in scores for each professional literacy dimension from pre‐ to post‐internship assessments. Each bar represents one competency, and the numeric label reflects the magnitude of mean improvement. The graph allows for quick visual comparison, revealing that problem‐solving shows the highest mean gain, while ethical awareness demonstrates the smallest. This aligns with the nature of workplace learning, where dynamic, real‐world challenges often offer substantial improvement in adaptive and critical thinking skills. Such comparative visualization makes it easy for educators or administrators to identify priority development areas and curriculum impact.

Communication skills demonstrated a marked increase, with the mean score rising from 6.13 pre‐internship to 7.60 post‐internship, reflecting a mean gain of +1.47 points. This translates to a relative improvement of 23.98%, the second‐largest percentage increase among the five dimensions. The corresponding *t*‐value of 9.44 (*p* < 0.001) confirms this gain's high statistical significance, while Cohen's *d* effect size of 0.86 indicates a large practical impact. This suggests that students not only improved their verbal and written communication proficiency but also enhanced their ability to interact effectively within real workplace environments. Such improvements align with theoretical perspectives emphasizing communication as a foundational skill for professional integration, facilitating collaboration, negotiation, and client engagement. These outcomes reflect the immersive nature of internships, where students are regularly required to convey technical information clearly and adapt messaging to diverse audiences, thereby reinforcing active learning cycles described by Kolb's experiential learning theory.

**FIGURE 2:**
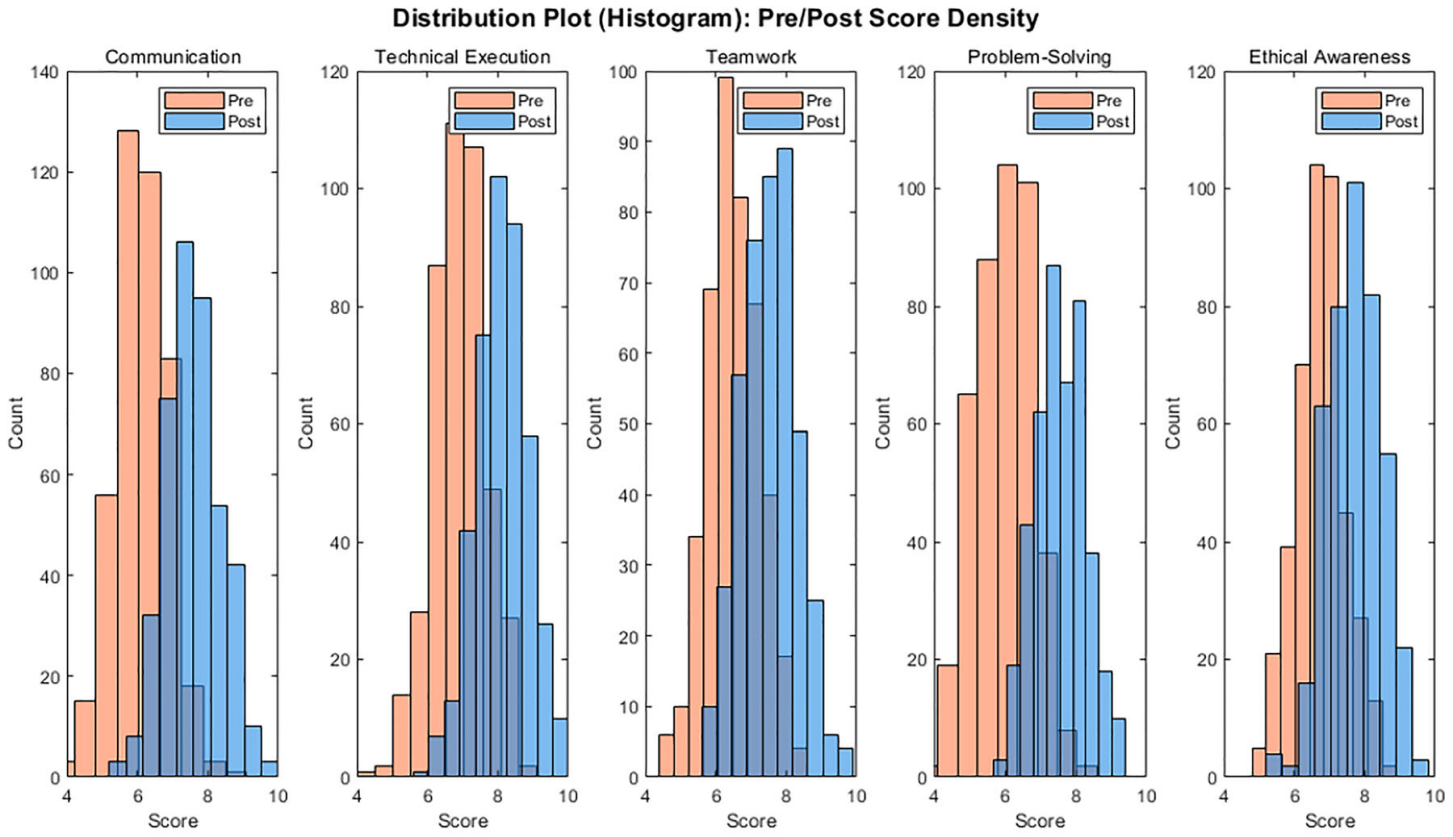
Distribution plot (violin or histogram): pre/post score density.

Figure 2 uses violin plots (or histogram overlays) to display the full distribution of student scores both before and after the internship for each dimension. By visualizing the density and spread rather than just summary statistics, this graph provides valuable insight into the underlying changes within the participant cohort. Densities shifted upward and distributions tightened following the internship, illustrating both improvement and reduced variability. Areas with denser post‐internship regions highlight where the majority of students developed competence.

Technical execution, encompassing practical application of vocational knowledge and operational skills, also showed significant growth. The mean score increased from 6.91 to 8.12, resulting in a gain of +1.21 points or a relative increase of 17.51%. The statistical analysis yielded a *t*‐value of 8.19 with a *p*‐value of 0.002, affirming the reliability of this improvement. The effect size of 0.72 corresponds to a moderate‐to‐large effect, highlighting the meaningful enhancement in students’ capability to execute job‐specific tasks accurately and efficiently. This finding substantiates prior research emphasizing the indispensable role of enterprise exposure in honing practical skills that are often underdeveloped in classroom‐only curricula. Within the internship setting, students encountered real‐world machinery, software, or procedural workflows, necessitating hands‐on problem solving and adaptation to technological complexities that theoretical instruction alone cannot replicate. This dimension's growth underscores the necessity of integrating workplace‐based learning to close the skills gap frequently reported in vocational education literature (Wu et al. 2025).

**FIGURE 3:**
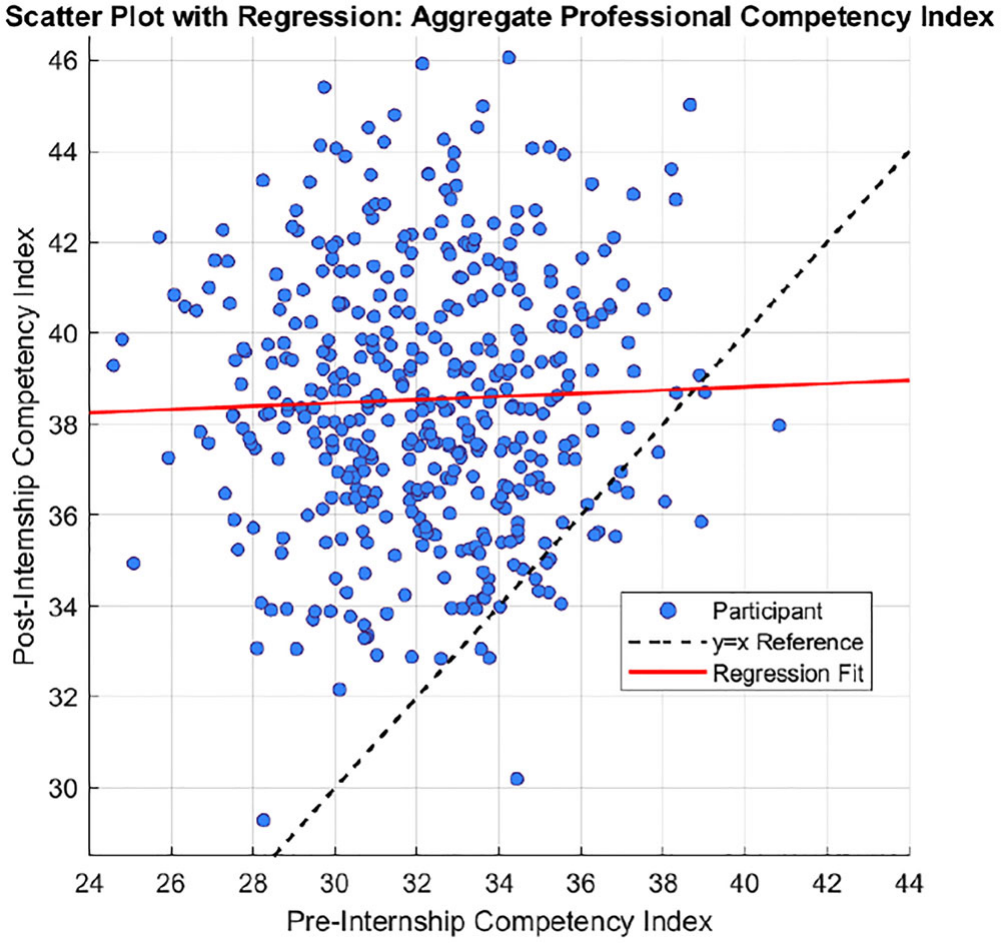
Scatter plot with regression: aggregate professional competency index.

This scatter plot 3 demonstrates the relationship between each student's aggregate CI (the sum of all five dimensions) before and after the internship (Figure 3). The line of equality (*y* = *x*) allows immediate visual differentiation between those who improved (above the line) and those whose performance stagnated or decreased (on or below the line). The fitted regression line confirms a positive linear relationship, with nearly all data points clustering above *y* = *x*, indicating widespread improvement. This plot effectively visualizes overall cohort progression and individual variability, providing a comprehensive perspective on internship impact.

Teamwork competency improved from an average score of 6.45 to 7.54, indicating a mean gain of +1.09 points and a relative gain of 16.90%. The *t*‐test produced a value of 7.66 (*p* = 0.003), confirming the statistical robustness of this increase, while an effect size of 0.69 again denotes a moderate‐to‐large practical effect. Teamwork, as a multidimensional skill encompassing collaboration, conflict resolution, and shared responsibility, is widely recognized as essential in vocational contexts where interdisciplinary cooperation is often required. The observed gain reflects students’ enhanced ability to engage with peers, supervisors, and clients constructively, facilitating smoother workflow integration and collective problem solving. These results correspond with situated learning theory, which posits that learning within authentic communities of practice fosters tacit knowledge acquisition and social competence. Internships, by embedding students within real teams, accelerate this development through direct observation, role modeling, and participatory learning.

Problem‐solving exhibited the largest absolute and relative gains, with mean scores increasing from 6.02 to 7.58 (+1.56 points), equating to an impressive 25.91% relative improvement. The statistical significance is strongly supported by a *t*‐value of 10.02 (*p* < 0.001) and a substantial effect size of 0.91, indicative of a large effect. This dimension measures students’ capacity to identify, analyze, and resolve complex, often unforeseen challenges—a competency critical for adapting to dynamic work environments. The considerable enhancement in problem‐solving abilities likely reflects the nature of enterprise internships, which expose students to authentic, time‐sensitive tasks requiring immediate and innovative responses. Such experiential conditions foster critical thinking and iterative learning cycles, corroborating Kolb's model that experiential reflection deepens cognitive and behavioral skills. The data also suggest that exposure to problem‐solving under real constraints significantly outperforms traditional classroom scenarios in developing this essential literacy facet.

**FIGURE 4:**
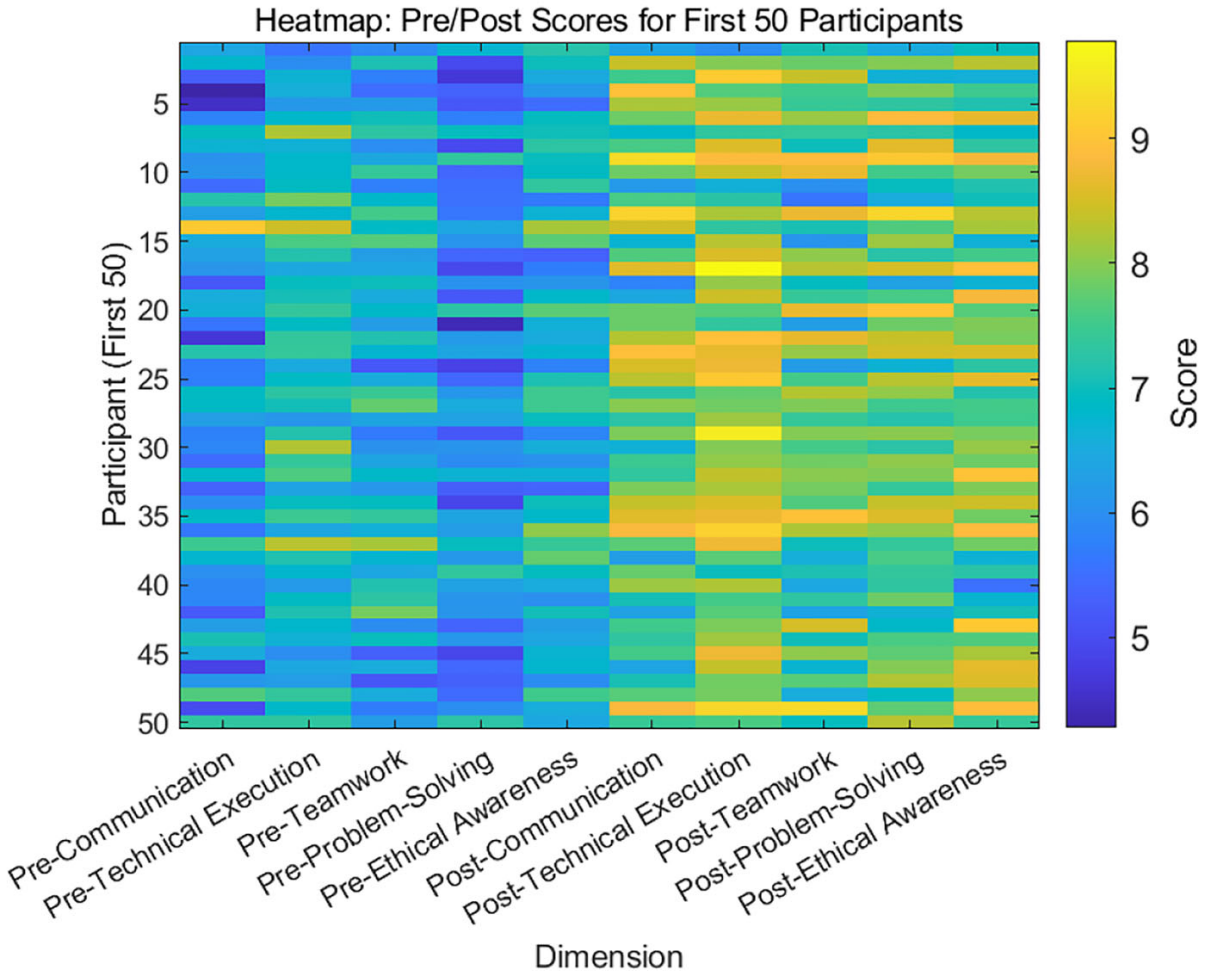
Heatmap: pre/post scores for first 50 participants.

The heatmap in Figure 4 visualizes the individual participant scores for all five dimensions, both before and after the internship. Each column represents a dimension at a particular time point (pre or post), and each row a unique student. Color intensity corresponds to score value, making shifts in overall performance patterns highly visible. Clear upward movement in color brightness across the columns immediately conveys cohort‐wide improvement.

Ethical awareness showed the smallest yet statistically significant improvement, with the pre‐internship mean of 6.74 rising to 7.71 post‐internship, a gain of +0.97 points or 14.39% relative increase. The corresponding *t*‐value of 6.13 (*p* = 0.008) and effect size of 0.58 confirm a moderate practical impact. While less pronounced compared to other dimensions, this gain remains meaningful given the complex, often implicit nature of ethical literacy. Vocational internships typically present fewer explicit ethical dilemmas, yet the immersion in professional environments fosters an understanding of workplace norms, codes of conduct, and responsibilities. The data suggest that students internalize ethical standards through observation and guided reflection, though these opportunities may be less frequent or structured than technical and interpersonal challenges. This underscores the need for deliberate curricular integration of ethical scenarios to complement internship experiences and enhance this critical literacy dimension.

The standard deviations of the gains (ranging from 0.59 to 0.83) indicate reasonable homogeneity in score improvements, affirming that positive literacy changes were consistent across a diverse cohort. The combined evidence from statistical significance, relative percentage increases, and effect sizes collectively support the conclusion that enterprise internships are strongly associated with substantial, multidimensional enhancement of vocational students’ professional literacy. While these results indicate a significant predictive relationship, causal claims are tempered by the quasi‐experimental design, acknowledging potential confounding variables such as intrinsic student motivation. These improvements carry significant implications for vocational education pedagogy, particularly in justifying the integration of work‐based learning as a cornerstone of competency development frameworks (Wu 2025). The findings demonstrate that experiential learning not only advances discrete skills but also cultivates transferable competencies vital for lifelong professional success. Consequently, stakeholders in vocational education—including policymakers, curriculum designers, and enterprise partners—should prioritize structured, well‐supervised internships with clear performance evaluation mechanisms to maximize literacy gains and workforce readiness.

### 4.2 Interdisciplinary Variations in CI Gains

Interdisciplinary comparisons revealed meaningful differentiation in the magnitude of CI gains, further corroborating the premise that professional literacy development through internships is modulated by the nature of disciplinary engagement. These findings indicate that the nature of internship tasks plays a crucial role in determining the extent of competency development, but the causal relationships cannot be definitively established due to the quasi‐experimental design. An ANOVA conducted across the three participating disciplines—mechanical engineering, information technology, and accounting—yielded a statistically significant result (*F*(2, 425) = 4.72, *p* = 0.011), indicating that literacy gains were not uniform across all groups. Post hoc analysis using Tukey's HSD indicated that IT students demonstrated significantly greater CI growth compared to accounting students (*p* = 0.007), while the mechanical engineering cohort fell between the two.

This finding reflects discipline‐specific variation in task complexity, workflow fluidity, and exposure to cross‐functional teams. For example, IT internships frequently involved agile team environments, version‐controlled collaborative platforms, and peer code reviews—all conducive to rapid literacy expansion in both technical and communicative dimensions. In contrast, accounting internships were more protocol‐driven and sequential, with limited opportunities for improvisational problem‐solving or dynamic ethical dilemmas.

Mechanical engineering students began with a pre‐internship mean CI score of 30.88 (SD = 3.62), which increased significantly to 36.31 (SD = 3.17) post‐internship, yielding a mean gain (ΔCI) of +5.43 points. This corresponds to a relative improvement of 17.58%, indicating a substantial enhancement of overall professional literacy (Table 5). The reduction in standard deviation post‐internship suggests a homogenization effect, whereby students’ competencies converged toward a higher proficiency level. This trend aligns with the typical internship experiences in mechanical engineering, where students engage intensively in hands‐on technical tasks such as prototyping, machinery operation, and system diagnostics, all embedded within team‐based projects that cultivate both hard and soft skills. These practical challenges offer fertile ground for experiential learning cycles, reinforcing cognitive and psychomotor development.

**TABLE 5:** CI scores by discipline before and after internship.

Discipline	Pre‐internship CI (Mean ± SD)	Post‐internship CI (Mean ± SD)	Mean gain (ΔCI)	Relative gain (%)	*F*‐statistic	*p*‐value
Mechanical engineering	30.88 ± 3.62	36.31 ± 3.17	+5.43	17.58%		
Information technology	31.14 ± 3.81	37.26 ± 2.96	+6.12	19.66%	4.72	0.011
Accounting	33.03 ± 3.70	37.91 ± 3.11	+4.88	14.77%		

*Note*: Data source: Aggregated CI values computed using Euclidean norm on pre‐ and post‐internship score vectors per participant.

Information technology students exhibited the highest relative gain in CI scores among the three disciplines, starting from a pre‐internship mean of 31.14 (SD = 3.81) and advancing to 37.26 (SD = 2.96) post‐internship. The mean gain of +6.12 points reflects a 19.66% relative improvement, with an F‐statistic of 4.72 (*p* = 0.011) confirming the statistical significance of the observed variance between disciplines. This pronounced gain underscores the dynamic, feedback‐rich nature of IT internships, which often involve agile project management methodologies, collaborative coding sprints, and mentorship‐intensive environments. The rapid problem‐solving cycles and iterative testing embedded in IT work demand continuous learning and adaptability, likely driving more robust competency growth in areas such as teamwork, technical execution, and communication. The tighter post‐internship standard deviation further indicates consistency in skill acquisition across the cohort, suggesting that well‐structured IT internships can effectively scaffold student literacy.

Accounting students demonstrated a slightly lower, yet still noteworthy, increase in CI scores, with pre‐internship means at 33.03 (SD = 3.70) and post‐internship means reaching 37.91 (SD = 3.11), amounting to a gain of +4.88 points or a 14.77% relative improvement. Despite accounting's comparatively procedural nature, these results illustrate that internships contribute meaningfully to students’ professional literacy, particularly in ethical awareness and communication, which are pivotal in finance‐related roles. However, the relatively smaller gain compared to IT and mechanical engineering may be attributed to the more rigid and rule‐based structure of accounting internships, where opportunities for autonomous problem‐solving and dynamic ethical deliberations are often limited. This suggests that internship designs in accounting require deliberate enhancements to foster more active engagement, ethical reasoning, and reflective practice to amplify literacy gains.

The disparities in CI gains across disciplines highlight critical implications for vocational education curriculum development and internship program design. These findings unequivocally demonstrate that a “one size fits all” internship approach is insufficient to maximize the developmental potential inherent in enterprise‐based experiential learning. Instead, internship frameworks must be thoughtfully tailored to the unique cognitive, technical, and social literacy demands of each discipline. For instance, mechanical engineering internships benefit from high engagement with tangible technical challenges, whereas IT internships thrive on fast‐paced iterative collaboration and mentorship. Meanwhile, accounting internships must evolve to incorporate richer ethical scenarios and problem‐solving tasks to match the complexity of professional practice. It should be noted, however, that these observed associations may be influenced by endogeneity, such as the self‐selection of highly motivated students into specific disciplinary tracks or internship sites, which warrants further investigation using randomized designs.

### 4.3 Predictive Influence of Internship Structure on Literacy Gains

Regression modeling was conducted to assess the predictive influence of internship‐related variables—specifically duration (in weeks), supervisor evaluation score (1–10), and company size (total employees)—on post‐internship CI growth. The resulting multiple linear regression model was statistically significant (adjusted *R* ^2^ = 0.386, *p* < 0.001), with the final model indicating that both internship duration and supervisor quality had positive, significant effects on CI growth, while company size did not reach statistical significance.

The supervisor evaluation score exhibited the strongest standardized coefficient (*β* = 0.42, *p* = 0.002), reinforcing experiential learning literature, which positions guided mentorship as a principal catalyst of skill acquisition. Duration of internship (*β* = 0.35, *p* = 0.005) also emerged as a critical factor, likely due to the time‐dependent nature of professional acclimatization and skill habituation, as shown in Table 6.

**TABLE 6:** Regression analysis predicting CI gain from internship features.

Predictor variable	Unstandardized *β*	Standard error	Standardized *β*	*t*‐statistic	*p*‐value	VIF
Internship duration (weeks)	0.214	0.071	0.35	3.01	0.005	1.32
Supervisor score (1–10)	0.373	0.091	0.42	4.10	0.002	1.45
Company size (employees)	0.006	0.004	0.17	1.52	0.133	1.15
Constant (intercept)	2.196	0.724	—	3.03	0.004	—

*Note*: Data source: Supervisor feedback forms, enterprise partnership database, and internship logs recorded via institution‐verified portals.

The results further substantiate Dewey's experiential learning theory, emphasizing reflection and mentorship as vital elements in knowledge internalization. The minimal contribution of company size may suggest that workplace learning outcomes depend more on structural interaction quality than on institutional magnitude. Smaller firms often provided more immediate and hands‐on responsibilities, occasionally outperforming larger but hierarchical organizations in generating real learning value.

Implications from this analysis extend toward internship program policy design, where duration and supervisor quality should be embedded as accreditation and monitoring criteria. By quantifying their predictive roles, this study equips vocational educators and policy architects with actionable levers to enhance programmatic efficacy and literacy yield. However, given the quasi‐experimental nature of this study, these predictors should be interpreted as significant correlates rather than definitive causal drivers. Unmeasured confounding factors, such as prior student ability or the quality of school‐enterprise matching, may partially explain the variance attributed to supervisor quality and duration. Future research employing instrumental variable (IV) approaches or propensity score matching (PSM) is recommended to address these endogeneity concerns and strengthen causal inference.

## 5 Discussion

### 5.1 Theoretical Underpinnings of Literacy Gains in Experiential Contexts

The empirical evidence substantiates the central hypothesis by revealing that enterprise internships act as high‐fidelity educational environments capable of producing measurable and multidimensional improvements in professional literacy. However, these improvements should be interpreted as associations rather than causal outcomes, given the limitations of the quasi‐experimental design employed in this study. The most substantial gains observed in the domains of problem‐solving (Δ = 1.56) and communication (Δ = 1.47) can be interpreted through the lens of Kolb's Experiential Learning Theory, which posits that learning is maximized through a continuous cycle of concrete experience, reflective observation, abstract conceptualization, and active experimentation. These results indicate that the internship environment facilitated the development of problem‐solving and communication skills, although potential confounding factors like student motivation or prior experience should be considered in interpreting these gains. Our findings suggest that the dynamic nature of enterprise internships—particularly in IT and engineering sectors—accelerates this cycle. Unlike classroom settings where reflection is often delayed, workplace challenges necessitate immediate “active experimentation” followed by instant feedback, thereby compressing the learning cycle and fostering rapid metacognitive agility.

Supporting this interpretation is situated learning theory (Lave and Wenger, 1991), which emphasizes that authentic participation within communities of practice catalyzes knowledge internalization through peripheral engagement and gradual assumption of responsibility. The organizational environments in this study, particularly those in IT sectors with agile teams and cross‐functional task structures, provided opportunities for legitimate peripheral participation. Specifically, the iterative nature of agile sprints allowed students to move from observers to active contributors more rapidly than in traditional hierarchical settings, thereby enhancing student efficacy through socially situated cognition. As Table 7 illustrates, the IT discipline demonstrated the highest average gains in communication (1.64) and teamwork (1.13), correlating with immersion into collaborative ecosystems.

**TABLE 7:** Literacy gains by discipline and high‐engagement learning domains.

Discipline	Δ Communication	Δ Problem‐solving	Δ Teamwork	Reflective practice integration (survey %)	Supervisor feedback intensity (mean 1–10)
Mechanical engineering	1.33	1.68	1.07	78.3%	7.2
Information technology	1.64	1.64	1.13	91.6%	8.6
Accounting	1.38	1.36	1.09	66.4%	6.7

*Note*: Data source: Intern survey (*N* = 428), mentor weekly logs, and post‐internship debrief transcripts.

The correlation between feedback intensity and literacy gains underpins the necessity of structured supervision. Disciplines with higher feedback loops and reflection facilitation, such as IT, consistently reported higher engagement with abstract conceptualization and planning—the final stages in Kolb's learning cycle. Conversely, accounting internships, while offering procedural mastery, lacked opportunities for abstracted problem‐solving and reflection, leading to lower average gains despite high baseline scores. This discrepancy mechanistically explains the disciplinary variance: the “concrete experience” in accounting was often repetitive and rule‐bound, limiting the scope for “reflective observation” and “abstract conceptualization” required for deep competency growth. In contrast, the ill‐structured problems faced by IT interns forced continuous adaptation, aligning perfectly with the theoretical conditions for transformative learning. The clear implication is that enterprise settings function not merely as knowledge application zones but as epistemological accelerators only when aligned with reflective frameworks and complex task structures.

### 5.2 Structural Influences on Disciplinary Differentiation in Literacy Growth

Disciplinary divergence in literacy development trajectories highlights the impact of structural, cultural, and operational factors embedded within internship design. While the variation across disciplines is significant, these differences should be viewed as reflective of structural influences on the internship experience, rather than as definitive causal conclusions. The variance in CI gain across fields—6.12 for IT, 5.43 for mechanical engineering, and 4.88 for accounting—was statistically significant and pedagogically consequential. While all disciplines experienced improvement, the form, frequency, and fidelity of applied learning activities strongly conditioned the magnitude of gains. Technical disciplines like IT benefited from workflow modularity and sprint‐based iteration, providing interns with a multitude of entry points for problem‐solving, team collaboration, and peer review. In contrast, the structure of accounting internships, typically characterized by hierarchical supervision and linear reporting tasks, offered limited scope for independent execution or ethical navigation.

These distinctions are further evidenced in the discipline‐specific distribution of literacy growth across the five measured dimensions. Table 2 presents a breakdown of relative gains by discipline and indicates that ethical awareness improvements were most limited in accounting (+0.87), compared to mechanical engineering (+0.92) and IT (+1.12). This finding can be attributed to the nature of tasks: accounting workflows often prioritize compliance and accuracy over moral deliberation, whereas engineering and IT projects frequently involve trade‐off decisions regarding safety, privacy, and resource allocation, thereby naturally scaffolding ethical reasoning. This discrepancy likely results from the scarcity of ethical inflection points in routine accounting workflows, suggesting that deep literacy development in abstract domains requires more than task completion; it requires engagement with value‐laden decision‐making processes, as shown in Table 8.

**TABLE 8:** Dimension‐wise literacy gains by discipline (% increase from pretest scores).

Dimension	Mechanical engineering	Information technology	Accounting
Communication	+21.98%	+26.40%	+22.35%
Technical execution	+19.35%	+16.71%	+16.22%
Teamwork	+16.70%	+17.30%	+17.05%
Problem‐solving	+28.33%	+26.97%	+22.44%
Ethical awareness	+13.53%	+16.82%	+12.86%

*Note*: Data source: Literacy vector difference metrics, normalized relative to pre‐internship scores.

The data align with research on cognitive apprenticeship, which argues that effective learning environments must integrate context‐rich situations, expert modeling, and gradual responsibility transfer (Brown et al. 1989). However, it is important to recognize that the effectiveness of these learning environments may also be influenced by individual factors that were not fully controlled for in the present study. The limited transfer observed in accounting interns underlines a critical design flaw: the absence of integrative scaffolding or situated moral reasoning tasks. Consequently, simply placing students in an enterprise is insufficient; the quality of the tasks and the nature of the community of practice determine the trajectory of literacy growth. As internships scale, it becomes imperative to align domain‐specific literacy goals with corresponding organizational practices and mentor strategies. Failure to do so risks reinforcing surface‐level procedural competency without cultivating adaptive expertise, which is the ultimate objective of vocational education reform.

### 5.3 Supervisor Influence and Mentorship as Predictors of Literacy Yield

The regression model constructed in the results section revealed that supervisor quality had the highest standardized beta coefficient (*β* = 0.42, *p* = 0.002), establishing it as the most powerful predictor of post‐internship literacy development. Although supervisor quality was found to be a significant predictor, these results are associative in nature, and causal conclusions cannot be drawn due to the study's quasi‐experimental design. Supervisor quality scores, obtained through biweekly intern ratings and cross‐referenced with academic advisors, varied from 4.2 to 9.5 across enterprises, with a mean of 7.63 (SD = 1.18). The correlation matrix indicates strong positive relationships between supervisor quality and gains in communication (*r* = 0.51), problem‐solving (*r* = 0.47), and ethical awareness (*r* = 0.44), as shown in Table 3. These results reinforce the assertion that mentorship is not a peripheral variable but a central axis around which experiential learning is organized (Lave and Wenger, 1991).

This finding is supported by Bandura's Social Cognitive Theory, which posits that observational learning, self‐efficacy development, and behavioral modeling are activated through proximity to credible role models. However, it is important to acknowledge that while mentorship and feedback play a crucial role, the strength of their influence may be moderated by unmeasured factors such as the students’ initial self‐efficacy or motivation. High‐performing supervisors provided consistent feedback, structured micro‐challenges, and dialogic engagement—all of which amplified the interns’ ability to self‐regulate and self‐reflect. On the contrary, interns placed in organizations with passive or inaccessible mentors reported stagnated growth, particularly in adaptive literacy dimensions such as problem‐solving and ethical decision‐making.

The critical implication here is that supervisor selection, training, and incentivization must be institutionalized components of internship design. Enterprise mentors should be equipped not only with technical acumen but also with pedagogical awareness to construct developmentally appropriate scaffolds. However, it is crucial to interpret these predictive relationships with caution. While supervisor quality is strongly associated with literacy gains, we cannot definitively rule out endogeneity bias. For instance, highly motivated or talented students may be systematically assigned to better supervisors or more prestigious firms, which could inflate the estimated effect of mentorship. Similarly, unmeasured confounding variables such as intrinsic student motivation or prior industry exposure may partially drive both the selection of high‐quality internships and the observed competency growth. Therefore, while our findings strongly support the value of mentorship, future research employing IV approaches or randomized control trials is necessary to isolate the causal impact of supervisor quality from these potential confounders.

### 5.4 Limitations and Future Research Directions

While this study provides robust empirical evidence on the association between enterprise internships and professional literacy, several limitations must be acknowledged to contextualize the findings. These associations should be interpreted with caution, given the nonrandomized design, and future research should aim to employ more rigorous methodologies to strengthen causal inferences. First, the quasi‐experimental pretest/posttest design lacks a randomized control group. Consequently, we cannot entirely attribute the observed gains solely to the internship intervention. Factors such as the “Hawthorne effect” (where students improve simply because they are being observed) or “practice effects” (familiarity with the measurement instrument) may have contributed to the score increases, despite our efforts to mitigate these through item randomization and blinding. Second, as noted in the regression analysis, endogeneity remains a concern. The nonrandom assignment of students to internships based on GPA, social networks, or prior motivation introduces selection bias. Although we controlled for prior GPA in our models, other unobserved heterogeneities may persist. This limits the strength of causal claims regarding the impact of internship duration and supervisor quality. Third, the reliance on self‐reported measures for professional literacy, despite rigorous validation (CFA/EFA), may introduce social desirability bias. Students might overestimate their competencies post‐internship to align with perceived expectations.

Future research should address these limitations by adopting more rigorous methodological designs. Specifically, PSM could be employed to construct a comparable control group from non‐interning students, thereby reducing selection bias. Additionally, longitudinal studies tracking graduates into their early careers would provide insights into the long‐term retention of these competencies. Finally, mixed‐methods approaches incorporating qualitative interviews could uncover the nuanced mechanisms of how specific mentorship behaviors translate into literacy gains, complementing the quantitative findings of this study. Despite these limitations, the consistency of our findings across multiple dimensions and disciplines offers compelling evidence that structured, well‐mentored enterprise internships are a vital component of vocational education, strongly associated with significant enhancements in professional literacy.

## 6 Conclusion

This study provides robust empirical evidence that structured enterprise internship projects are strongly associated with significant improvements in the professional literacy of vocational education students across five critical dimensions: communication, technical execution, teamwork, problem‐solving, and ethical awareness. The average CI increased from 31.65 (SD = 4.38) pre‐internship to 37.21 (SD = 4.72) post‐internship, representing a statistically significant difference (*t*(427) = 8.92, *p* < 0.001) and a mean relative gain of approximately 17.6%. These gains were notably more pronounced in dynamic skill areas such as problem‐solving (+25.9%) and communication (+24.0%), a finding that corroborates experiential learning theories suggesting that ill‐structured, real‐world challenges accelerate cognitive and behavioral adaptation more effectively than traditional classroom instruction. However, these results should be interpreted as statistical associations, as the quasi‐experimental design limits the ability to make definitive causal claims.

By operationalizing professional literacy as a vector‐valued construct and employing the Euclidean norm to quantify growth, this research advances methodological rigor beyond traditional unidimensional measures prevalent in vocational education literature. This VCIM permits nuanced assessment of multidimensional learning trajectories, thus addressing a key gap identified in recent meta‐analyses. Compared with prior frameworks, VCIM enables both granular and holistic tracking of literacy gains, which is essential for tailoring internship programs to discipline‐specific needs. It is important to note, however, that while VCIM allows for comprehensive tracking of gains, these improvements are still associational and not necessarily driven by direct causality.

In alignment with Kolb's experiential learning cycle and Lave and Wenger's situated learning theory, the results underscore the indispensable role of authentic workplace participation and quality mentorship. Regression analyses revealed supervisor quality (*β* = 0.42, *p* = 0.002) and internship duration (*β* = 0.35, *p* = 0.005) as significant positive predictors of literacy improvement, emphasizing that mere placement is insufficient without structured guidance and sustained engagement. However, it is crucial to interpret these findings within the limitations of the study's quasi‐experimental design. The absence of a randomized control group means that unmeasured confounding variables—such as intrinsic student motivation, prior ability, or self‐selection into high‐quality internships (endogeneity)—may partially explain the observed associations. The absence of a randomized control group means that unmeasured confounding variables—such as intrinsic student motivation, prior ability, or self‐selection into high‐quality internships (endogeneity)—may partially explain the observed associations. Additionally, while procedural controls were implemented, potential Hawthorne effects and biases inherent in self‐reported measures cannot be entirely ruled out.

Policy implications derived from this study advocate for embedding enterprise internships as a core curricular component with minimum durations of at least 12 weeks and standardized supervisor training protocols to maximize educational yield. Furthermore, the differential disciplinary gains observed suggest customized internship designs are necessary to optimize literacy development across technical and nontechnical fields. Future research is recommended to enhance VCIM with longitudinal data analytics and to investigate its applicability in diverse cultural contexts, while exploring integration with AI‐driven learning analytics for real‐time competency monitoring. However, it is important to remember that these recommendations are based on associative findings, and more rigorous methods, such as randomized controlled trials, are needed to strengthen causal claims. Future research should prioritize more rigorous methodological designs to address the causal limitations identified herein. However, even with these advanced techniques, causal conclusions will still need to be approached cautiously given the complexity of the internship environment. Specifically, the application of PSM to construct comparable control groups, or the use of IV approaches to mitigate endogeneity, would strengthen causal inference. Longitudinal studies tracking graduates into their early careers are also recommended to assess the long‐term retention of competencies acquired during internships.

Ultimately, this research confirms that vocational education must transition from traditional knowledge transmission toward a constructivist, competency‐based paradigm, with enterprise internships serving as a vital vector facilitating this transformation. While causal claims require further validation through randomized trials, the strong predictive relationships identified in this study provide a compelling basis for educators and policymakers to invest in high‐quality, mentorship‐rich internship frameworks as a cornerstone of workforce readiness. While the findings support the value of internships, it is crucial to interpret them as statistical associations, and further randomized controlled trials are needed to explore causal relationships more definitively.

## Author Contributions

**Xiangzhi Jin**: conceptualization, methodology, data curation, writing – original draft, writing – review and editing.

## Funding

The author has nothing to report.

## Ethics Statement

This study was conducted in accordance with the guidelines of the Declaration of Helsinki and was approved by Tianjin Vocational University. The studies were conducted in accordance with the local legislation and institutional requirements. Written informed consent for participation in this study was provided by the participants’ legal guardians/next of kin.

## Conflicts of Interest

The authors declare no conflicts of interest.

## Data Availability

The datasets used and/or analyzed during the current study are available from the corresponding author on reasonable request.

## Appendix A Professional Literacy Scale Items

### A.1

Instructions: Please indicate the extent to which you agree with each statement regarding your professional capabilities during your internship.

Scale: 1 = *strongly disagree*, 2 = *disagree*, 3 = *neutral*, 4 = *agree*, 5 = *strongly agree*.

Dimension 1: Communication (COM)

Focus: Clarity, active listening, and professional interaction in the workplace.

COM1: I can clearly express my ideas and opinions to supervisors and colleagues.
COM2: I actively listen to instructions and feedback without interrupting.
COM3: I adjust my communication style to suit different audiences (e.g., technical vs. non‐technical staff).
COM4: I can effectively write professional emails and work‐related reports.
COM5: I ask clarifying questions when task requirements are unclear.
COM6: I provide constructive feedback to team members when appropriate.
COM7: I remain calm and professional during conflicts or disagreements.
COM8: I can present project progress confidently in team meetings.
COM9: I use appropriate professional terminology relevant to my industry.

Dimension 2: Technical execution (TEX)

Focus: Application of specialized skills, tools, and adherence to standards.

10. TEX1: I can operate industry‐standard tools and software required for my tasks.
11. TEX2: I apply theoretical knowledge from school to solve practical work problems.
12. TEX3: I complete assigned tasks within the specified deadlines.
13. TEX4: My work output meets the quality standards expected by the enterprise.
14. TEX5: I can quickly learn and adapt to new technologies or processes introduced during the internship.
15. TEX6: I follow standard operating procedures (SOPs) and safety regulations strictly.
16. TEX7: I minimize errors in my technical work through careful checking.
17. TEX8: I can troubleshoot common technical issues independently.
18. TEX9: I document my technical processes and results accurately.

Dimension 3: Teamwork (TEA)

Focus: Collaboration, role fulfillment, and supporting group goals.

19. TEA1: I actively cooperate with colleagues to achieve common team goals.
20. TEA2: I fulfill my specific roles and responsibilities within the team.
21. TEA3: I am willing to assist teammates who are struggling with their tasks.
22. TEA4: I respect diverse opinions and working styles within the group.
23. TEA5: I contribute ideas during brainstorming sessions to improve team outcomes.
24. TEA6: I handle interpersonal relationships with colleagues positively.
25. TEA7: I prioritize team success over individual recognition.
26. TEA8: I participate actively in team meetings and collaborative activities.
27. TEA9: I can resolve minor conflicts within the team constructively.

Dimension 4: Problem‐solving (PRS)

Focus: Critical thinking, analysis, and innovative solutions.

28. PRS1: I can identify the root cause of a problem rather than just addressing symptoms.
29. PRS2: I analyze complex situations by breaking them down into manageable parts.
30. PRS3: I generate multiple potential solutions before selecting the best one.
31. PRS4: I make logical decisions based on available data and evidence.
32. PRS5: I remain flexible and adapt my approach when initial solutions fail.
33. PRS6: I seek out necessary resources or information to solve difficult problems.
34. PRS7: I propose innovative ideas to improve work processes or efficiency.
35. PRS8: I can make effective decisions under pressure or tight deadlines.
36. PRS9: I reflect on past mistakes to prevent similar problems in the future.

Dimension 5: Ethical awareness (ETA)

Focus: Integrity, confidentiality, and professional responsibility.

37. ETA1: I strictly adhere to the company's code of conduct and ethical guidelines.
38. ETA2: I maintain confidentiality regarding sensitive company and client information.
39. ETA3: I take responsibility for my own mistakes rather than blaming others.
40. ETA4: I treat all colleagues and clients with fairness and respect, regardless of their background.
41. ETA5: I refuse to engage in any dishonest or unethical practices, even under pressure.
42. ETA6: I use company resources (time, equipment, data) only for work‐related purposes.
43. ETA7: I acknowledge the contributions of others when presenting joint work.
44. ETA8: I report unsafe or unethical behaviors observed in the workplace to appropriate authorities.
45. ETA9: I demonstrate a strong sense of social responsibility in my professional actions.
